# Lung cancer stage at diagnosis: Individual associations in the prospective VITamins and lifestyle (VITAL) cohort

**DOI:** 10.1186/1471-2407-11-228

**Published:** 2011-06-07

**Authors:** Christopher G Slatore, Michael K Gould, David H Au, Mark E Deffebach, Emily White

**Affiliations:** 1 Portland VA Medical Center, Health Services Research & Development; Portland, OR, USA; 2 Oregon Health & Sciences University, Division of Pulmonary and Critical Care Medicine; Portland, OR, USA; 3 Keck School of Medicine of the University of Southern California, Division of Pulmonary and Critical Care Medicine; Los Angeles, CA, USA; 4 VA Puget Sound Health Care System, Health Services Research and Development; Seattle, WA, USA; 5 University of Washington, Division of Pulmonary and Critical Care; Seattle, WA, USA; 6 Portland VA Medical Center; Portland, OR, USA; 7 University of Washington, Department of Epidemiology; Seattle, WA, USA; 8 Fred Hutchinson Cancer Research Center, Cancer Prevention Program; Seattle, WA, USA

## Abstract

**Background:**

Lung cancer is the leading cause of cancer death in the United States. Identifying factors associated with stage of diagnosis can improve our understanding of biologic and behavioral pathways of lung cancer development and detection. We used data from a prospective cohort study to evaluate associations of demographic, health history, and health behaviors with early versus late stage at diagnosis of non-small cell lung cancer (NSCLC).

**Methods:**

We calculated odds ratios (ORs) for the association of patient-level characteristics with advanced stage of diagnosis for NSCLC. The OR's were then adjusted for age, gender, race/ethnicity, smoking status, income, education, chronic obstructive pulmonary disease, and a comorbidity index.

**Results:**

We identified 612 cases of NSCLC among 77,719 adults, aged 50 to 76 years from Washington State recruited in 2000-2002, with followup through December 2007. In univariate analyses, subjects who quit smoking <10 years (OR 2.56, 95% CI 1.17 - 5.60) and were college graduates (OR 1.67, 95% CI, 1.00 - 2.76) had increased risks of being diagnosed with advanced stage NSCLC, compared to never smokers and non-college graduates, respectively. Receipt of sigmoidoscopy/colonoscopy, compared to no receipt, was associated with a decreased risk of advanced stage (OR 0.65, 95% CI, 0.43 - 0.99). The adjusted OR for receipt of sigmoidoscopy/colonoscopy was 0.55 (95% CI, 0.36 - 0.86). There was evidence that increasing the number of screening activities was associated with a decreased risk of advanced stage NSCLC (P for trend = 0.049).

**Conclusions:**

Smoking status, education, and a screening activity were associated with stage at diagnosis of NSCLC. These results may guide future studies of the underlying mechanisms that influence how NSCLC is detected and diagnosed.

## Background

Lung cancer is the second most common cancer amongst men and women and the leading cause of cancer-related mortality [1]. Lung cancer mortality is high since it is often detected after development of late stage disease [1]. Accordingly, there is substantial interest in methods to prevent lung cancer, inhibit or slow down its growth and progression, and to detect early stage lung cancer through the use of imaging or biomarker modalities [2,3].

Cancer is detected based on symptoms, incidental findings, or active screening [4]. Accordingly, mechanisms that alter the development, perception, or reporting of symptoms, alter the likelihood of finding cancer incidentally, and/or alter the likelihood of screening may impact the stage of diagnosis. Identifying individual factors associated with stage of diagnosis can improve our understanding of biologic and behavioral pathways of lung cancer development and detection.

Individual predictors of lung cancer diagnosis stage have not been well studied. We used the data from a prospective cohort study (the Vitamins and Lifestyle (VITAL) study) to evaluate associations of demographic, health history, and health behaviors with early versus late stage at diagnosis of non-small cell lung cancer (NSCLC). Because of a suggestion that lung cancer screening is ongoing [5], we hypothesized that other screening behaviors might be associated with a decreased risk of an advanced stage at diagnosis.

## Methods

### Subjects

The VITAL study is a prospective cohort of community-dwelling adults [6]. Women and men were eligible if they were aged 50 to 76 and lived in the area covered by the Seattle-Puget Sound Surveillance, Epidemiology, and End Results (SEER) cancer registry. Using a commercial list, we mailed 364,418 questionnaires from October 2000 to December 2002. 77,719 (21.3%) participants returned questionnaires and passed eligibility and quality control checks. Baseline data were obtained from a sex-specific 24-page self-administered questionnaire that included items on medication use, diet, medical history, personal characteristics, and cancer risk factors. The Institutional Review Board of the Fred Hutchinson Cancer Research Center approved the protocol.

Participants were followed for lung cancer occurring from baseline through December 31, 2007, by linking the cohort to the Seattle-Puget Sound SEER registry. Cases and information on tumor characteristics, including histology and stage at diagnosis, are ascertained through all hospitals in the area, offices of pathologists, oncologists, and radiotherapists, and from state death certificates. The SEER registry has been shown to have accurate and complete data collection [7] and is reliable for lung cancer histology [8]. If a subject had multiple diagnoses of lung cancer, we used the stage of the first primary diagnosis.

We excluded participants with a previous diagnosis of lung cancer reported at baseline (n = 376) or for whom this datum was missing (n = 211). We also excluded subjects whose lung cancer was identified on a death certificate only or whose lung cancer morphology was classified as lymphoma (n = 10). We elected to only include cases of NSCLC since growth rates, staging, and symptomatology markedly differ from small cell lung cancer. After these exclusions, 612 participants developed NSCLC within a mean followup time of 5.9 years (SD 1.2 years).

### Outcome Assessment: Stage at Diagnosis

Based on SEER data, we dichotomized stage at diagnosis into in-situ and local (early stage) versus regional, distant, or unknown stage (advanced stage). Unknown stage was combined with the latter group because it has a comparable survival rate [1].

### Covariates

#### Sociodemographic & Health History

Subjects reported demographic and socioeconomic factors that included age, race/ethnicity, marital status, and education. Self-report of physician-diagnosed chronic obstructive pulmonary disease (COPD), including emphysema and chronic bronchitis, and previous history of cancer were recorded. We categorized family history of lung cancer as none or at least one first degree relative with lung cancer. A comorbidity scale was created based on self-report of the following conditions, categorized as yes or no for each response: coronary artery disease, heart failure, stroke, chronic pulmonary disease, rheumatoid arthritis, cirrhosis or other chronic liver disease, kidney disease (other than kidney stones), diabetes, and history of cancer other than non-melanotic skin cancer.

Subjects were asked detailed questions about their exercise habits. Exercise is calculated as usual metabolic equivalent of task (MET) hours per week for each activity averaged over the previous 10 years as follows: [Frequency of activity per week * minutes per session * years in the past 10 * MET for that activity] / [(60 minutes/hour) * 10 years] [9]. We then summed the MET hours for all activities to calculate total 10-year average MET hours per week. We categorized exercise into quartiles. Body mass index (BMI) was calculated from the respondent's self-reported current weight and height, measured as kg/m^2^, and categorized. Daily servings of fruit were assessed by a food frequency questionnaire (FFQ) that was an adaptation of FFQ's developed for the Women's Health Initiative and other studies [10-12], with the addition of highly supplemented foods. The measurement properties of earlier versions of this questionnaire have been published [10].

#### Tobacco

Smokers were defined as individuals who smoked at least one cigarette per day for at least a year. Smoking status was classified as never, current, quit 10 years or more or quit less than 10 years ago, at the date of questionnaire completion. Duration of smoking was estimated by the reported number of years smoked, intensity by the usual number of cigarettes smoked per day, and pack-years was computed as years smoked × cigarettes per day/20.

#### Screening Activities

All subjects were asked if they had a sigmoidoscopy or colonoscopy in the ten years prior to baseline. Men reported if they had a prostate specific antigen (PSA) test in the two years prior to baseline. Women reported if they had a mammogram in the two years prior to baseline.

### Statistical Analysis

All statistical analyses were performed using Stata SE-11 (StataCorp, College Station, TX). For the univariate analyses, the association between each factor and early versus advanced stage at diagnosis was measured through logistic regression using robust standard errors. To evaluate our primary hypothesis that screening activities would be associated with stage at diagnosis, we used multivariable adjusted logistic regression using robust standard errors. *A priori*, we decided to adjust for age at baseline (continuous), gender, smoking status (never, current, quit < 10 years ago, quit ≥ 10 years ago), income (classified as greater or less than $40,000/year and missing), education (dichotomized as greater or less than college graduate), race/ethnicity (dichotomized as white versus other), comorbidity (modeled continuously), and self-reported COPD (dichotomized as yes versus no). Overall, there was less than 5% missing information for all variables except income (21% missing this information) and BMI (6% missing this information). P values less than 0.05 were considered statistically significant.

We evaluated whether other factors individually confounded the association of screening activities with NSCLC stage at diagnosis in the adjusted model. These included: years of smoking, packyears, history of cancer, exercise, BMI, marital status, and servings of fruit. None changed the point estimates of the screening activity variables by more than 10% or the 5% level of significance so they were not included in the final model. We performed sensitivity analyses by not including in-situ and unknown stages in the outcome. As not including these subjects in the screening analyses did not substantively change the OR's, we decided to include all stages in the final model.

We examined whether the association between sigmoidoscopy/colonoscopy receipt and NSCLC stage at diagnosis differed by age, sex, and smoking status. These models were adjusted as above with the exception of not including age, sex, or smoking status, respectively, in the stratified models. Since there were few never smokers who developed lung cancer, we did not include this group in the stratified smoking status analyses. Likelihood ratio tests were conducted to assess the interaction between sigmoidoscopy/colonoscopy and the subgroups. P values for interaction were obtained to compare the fit of the models with the interaction terms and without them.

### Role of the Funding Source

This work was completed with grant support from the CHEST Foundation of the American College of Chest Physicians and the LUNGevity Foundation to C.S. This work was also supported by the National Institutes of Health [K05CA154337 to EW]. Drs. Slatore, Au, and Deffebach were supported by the Department of Veterans Affairs. This study is the result of work supported by resources from the Portland VA Medical Center, Portland, OR, and VA Puget Sound Health Care System, Seattle, Washington. The study sponsors had no role in the conduct of the study, in the collection, management, analysis, or interpretation of data, or in the preparation, review, or approval of the manuscript.

## Results

Of the 612 cases of NSCLC, there were 280 cases of adenocarcinoma (45.6%), 137 of squamous cell (22.4%), 18 of large cell (2.9%) and 177 of NSCLC, not otherwise specified (28.9%). One subject (0.2%) was diagnosed with an in situ stage at diagnosis, 131 (21.4%) local, 159 (26.0%) regional, 311 (50.8%) distant, and 10 (1.6%) had an unknown stage.

When stratified by the stage at diagnosis, subjects were similar in terms of age, race/ethnicity, smoking duration/intensity, and history of cancer (Table 1). Women and never smokers were less likely to be diagnosed with advanced stage. Former smokers who had quit < 10 years were more often diagnosed with advanced stage disease. College graduates were more often diagnosed with advanced stage disease.

**Table 1:** Characteristics of cohort with non-small cell lung cancer, stratified by stage at diagnosis

Characteristic	Early Stage In-Situ/Local N = 132 N or Mean (% or SD)	Advanced Stage Regional/Distant/Unknown N = 480 N or Mean (% or SD)
**Demographics**		
Age (years)	67.0 (6.5)	67.0 (6.5)
Women	70 (53.0%)	209 (43.5%)
Non-White Race/Ethnicity	6 (4.6%)	27 (5.6%)
**Smoking**		
Years of Smoking (years)*	32.4 (15.0)	33.8 (13.6)
Pack Years (years)*	40.3 (26.8)	44.3 (28.5)
**Smoking Status**		
Never smoker	16 (12.1%)	32 (6.7%)
Current smoker	39 (29.6%)	141 (29.4%)
Former, Quit <10 yr	18 (13.6%)	92 (19.2%)
Former, Quit ≥10 yr	58 (43.9%)	211 (44.0%)
**Socioeconomic**		
College Graduate or Higher	22 (16.7%)	121 (25.2%)
Income > $40,000/year	48 (36.4%)	189 (39.4%)
Currently Married/Partner	94 (71.2%)	327 (68.1%)
**Medical History**		
COPD	23 (17.4%)	70 (14.6%)
History of Cancer	28 (21.2%)	102 (21.3%)
Family History Lung Cancer**	30 (22.7%)	89 (18.5%)
Comorbid Disease*** (1 or more)	74 (56.1%)	273 (56.9%)
Exercise (Highest Quartile)****	22 (16.7%)	89 (18.5%)
**BMI Category (kg/m^2^)**		
Normal (18.5 - 24.9)	41 (31.1%)	164 (34.2%)
Underweight (<18.5)	4 (3.0%)	8 (1.7%)
Overweight (25 - 29.9)	55 (41.7%)	191 (39.8%)
Obese (≥30)	23 (17.4%)	89 (18.5%)
**Screening Activities**		
Sigmoidoscopy/Colonoscopy	89 (67.4%)	275 (57.3%)
PSA (men only)	45 (72.6%)	187 (69.0%)
Mammogram (women only)	61 (87.1%)	191 (91.4%)
Screening (at least one screening study)	118 (89.4%)	419 (87.3%)

Note: percentages are of total and may not sum to 100% secondary to rounding and missing information

Abbreviations

BMI: Body Mass Index

COPD: Chronic Obstructive Pulmonary Disease

*among current or former smokers

** ≥1 1^st ^degree relative with lung cancer

***See definition in text

**** usual MET hours per week for each activity averaged over the previous 10 years

In the univariate analyses of the association of potential factors with stage at diagnosis, subjects who quit smoking <10 years (OR 2.56, 95% CI 1.17 - 5.60, P = 0.019) and were college graduates (OR 1.66, 95% CI, 1.00 - 2.76, P = 0.048) had increased risks of being diagnosed with advanced stage NSCLC, compared to never smokers and non-college graduates, respectively (Table 2). Receipt of sigmoidoscopy/colonoscopy (OR 0.65, 95% CI, 0.43 - 0.99, P = 0.043) was associated with a decreased risk of advanced stage. Women were less likely to be diagnosed with advanced stage (OR 0.68, 95% CI, 0.46 - 1.01, P = 0.053) though this association was not significant.

**Table 2:** Univariate Odds Ratios (OR's) for associations with advanced stage at diagnosis of non-small cell lung cancer

Variable	Advanced Stage Regional/Distant/Unknown	
	OR	95% CI
**Demographics**		
Age (per year)	1.00	(0.98 - 1.03)
Women	0.68	(0.46 - 1.01)
Non-White Race/Ethnicity	1.24	(0.50 - 3.07)
**Smoking**		
Years of Smoking (10 year increments)*	1.07	(0.93 - 1.23)
Pack Years (10 year increments)*	1.05	(0.98 - 1.13)
**Smoking Status**		
Never smoker	Ref	
Current smoker	1.81	(0.90 - 3.63)
Former, Quit <10 yr	2.56	(1.17 - 5.60)
Former, Quit ≥10 yr	1.82	(0.93 - 3.55)
**Socioeconomic**		
College Graduate or Higher	1.66	(1.00 - 2.76)
Income >$40,000/year	1.04	(0.67 - 1.62)
Currently Married or with Partner	0.77	(0.49 - 1.21)
**Medical History**		
COPD	0.81	(0.48 - 1.35)
History of Cancer	1.00	(0.62 - 1.60)
Family History Lung Cancer**	0.79	(0.49 - 1.26)
Comorbid Disease*** (1 or more)	1.03	(0.69 - 1.52)
Exercise (Highest Quartile)****	1.15	(0.69 - 1.93)
**BMI Category (kg/m^2^)**		
Normal (18.5 - 24.9)	Ref	
Underweight (<18.5)	0.50	(0.14 - 1.74)
Overweight (25 - 29.9)	0.87	(0.55 - 1.37)
Obese (≥30)	0.97	(0.55 - 1.72)
Missing	0.78	(0.34 - 1.78)
**Screening Activities**		
Sigmoidoscopy/Colonoscopy	0.65	(0.43 - 0.99)
PSA (men only)	0.93	(0.50 - 1.73)
Mammogram (women only)	1.47	(0.61 - 3.59)
Screening (at least one screening study)	0.81	(0.43 - 1.53)

*among current or former smokers

** ≥1 1^st ^degree relative with lung cancer

***See definition in text

**** usual MET hours per week for each activity averaged over the previous 10 years

We then examined the adjusted associations between screening activities and stage at diagnosis (Table 3). Receipt of sigmoidoscopy/colonoscopy was associated with a decreased risk of being diagnosed with advanced NSCLC (OR 0.55, 95% CI, 0.36 - 0.86, P < 0.01). Neither PSA testing or mammography were associated with stage at diagnosis. There was evidence that increasing the number of screening activities was associated with a decreased risk of advanced stage NSCLC (P for trend = 0.049).

**Table 3:** Adjusted Odds Ratios (OR's) for association of screening activities with advanced stage at diagnosis of non-small cell lung cancer

Variable	Advanced Stage Regional/Distant/Unknown	
	OR*	95% CI
**Screening Activity**		
Sigmoidoscopy/Colonoscopy	0.55	(0.36 - 0.86)
PSA	0.93	(0.50 - 1.74)
Mammogram	1.55	(0.55 - 4.39)
**Number of Screening Activities**		
One	1.00	(0.48 - 2.07)
Two	0.62	(0.31 - 1.26)
P for trend	0.049	

*Adjusted for age, gender, smoking status, income (included missing as a category), education, race/ethnicity, comorbidity, & self-reported COPD

We stratified the results of the sigmoidoscopy/colonoscopy receipt and the stage at diagnosis associations by age, gender, and smoking status (Table 4). There was no evidence of effect modification for any of these variables.

**Table 4:** Adjusted Odds Ratios (OR's) for association of sigmoidoscopy/colonoscopy receipt with advanced stage at diagnosis of non-small lung cancer, stratified by age, sex, and smoking status

Sigmoidoscopy/Colonoscopy	Advanced Stage Regional/Distant/Unknown	
	OR	95% CI
Age*		
Age <65	0.70	(0.32 - 1.55)
Age ≥65	0.53	(0.30 - 0.94)
P for interaction	0.68	
Gender**		
Women	0.59	(0.31 - 1.14)
Men	0.52	(0.27 - 1.00)
P for interaction	0.63	
Smoking Status***		
Current smoker	0.65	(0.30 - 1.42)
Former, Quit <10 yr	0.19	(0.04 - 0.87)
Former, Quit ≥10 yr	0.56	(0.28 - 1.16)
P for interaction	0.46	

*Adjusted for gender, smoking status, income (included missing as a category), education, race/ethnicity, comorbidity, & self-reported emphysema

**Adjusted for age, smoking status, income (included missing as a category), education, race/ethnicity, comorbidity, & self-reported emphysema

***Adjusted for age, gender, income (included missing as a category), education, race/ethnicity, comorbidity, & self-reported COPD

## Discussion

This study found few demographic or socioeconomic factors, health history, or health behaviors that were associated with stage of diagnosis of non-small cell lung cancer. Subjects who quit smoking <10 years and were college graduates had increased risks of being diagnosed with advanced stage NSCLC, compared to never smokers and non-college graduates, respectively, whereas sigmoidoscopy/colonoscopy receipt was associated with a decreased risk. These associations should not be construed as causal but may be important factors in the development and detection of NSCLC.

Cancer is detected based on symptoms, incidental findings, or active screening and differences in stage at diagnosis must operate via one or more of these mechanisms [4]. In terms of symptoms, individuals may delay care despite the presence of symptoms and present with advanced stages. Patients and clinicians may delay symptomatic care for several reasons that include health beliefs, limited access to healthcare, and/or competing causes (e.g. attributing a cough in a smoker to a benign cause). Also, factors that alter symptoms for a particular stage could be associated with a differential stage at diagnosis (e.g. causing hemoptysis at an earlier stage). Second, lung cancer is commonly found incidentally on imaging studies [13]. Individuals with decreased access to care may have fewer opportunities to have asymptomatic, early stage tumors discovered incidentally [14-17]. Comorbid diseases might also alter the likelihood of incidental findings (e.g. a patient with congestive heart failure is found to have an incidental early stage tumor on a chest x-ray). A third factor is active screening to identify asymptomatic cases.

Stage at diagnosis is a strong predictor of lung cancer mortality [18] so identifying individual factors associated with stage at diagnosis is important for several reasons. First, identification may aid the development of disease progression biomarkers through better understanding of potential confounders [19]. Second, these factors may be in the causal pathway for previously studied factors, including race/ethnicity [16], census tract-level socioeconomic status [14], rural versus urban location [15], and insurance status [17], that have been associated with lung cancer care disparities. Finally, it is likely that some lung cancers grow too slowly to cause death [20-22]. Understanding factors associated with slow-growing tumors, those with a higher chance of being found incidentally at earlier stages, may aid research into underlying mechanisms of tumor growth and development.

It is intriguing that a screening behavior was associated with lower risk of an advanced stage at diagnosis after adjusting for many potential confounders such as access to care variables, comorbid diseases, and other health behaviors. In addition, we compared in-situ and local disease versus advanced disease; local stages are rarely symptomatic [21] so factors that are mediated by symptoms are unlikely to explain our findings. Smoking status has not been evaluated in terms of its association with stage of diagnosis but a recent study from Sweden did not find an association with education status [23]. Patients who received a sigmoidoscopy/colonoscopy as of 2002, and their clinicians, may have been "early adopters" of screening as there was no solid evidence of its benefit for preventing colon cancer mortality at that time [24]. At the time of questionnaire administration lung cancer screening was not recommended [25]. Computed tomography (CT) may soon have a role in early detection based on a preliminary report from the National Lung Cancer Screening Trial that reports to show a 20% decrease in lung cancer mortality in the screened group [26]. Even before the results of this study became available, some experts and advocacy groups recommend that high risk groups discuss the utility of screening with their clinicians [27-29]. Similar to the widespread adoption of prostate cancer screening prior to evidence of its benefit [30,31], lung cancer screening was advocated by some groups without knowledge of its risks and benefits [32]. Patients may currently undergo CT screening for lung cancer based on their own or clinician beliefs about its efficacy [5,33,34] and our results raise this possibility as well. Understanding screening behaviors prior to the implementation of widespread recommendations for CT screening will assist evaluation of its implementation.

Our study has several strengths. We used a large, prospective, population-based cohort study design. We controlled for comorbid disease and factors associated with access to care. Although we were unable to directly adjust for insurance status, we adjusted for age in the primary analysis and did not observe effect modification for subjects over and under age 65, the age where there is essentially universal coverage through Medicare. Finally, the SEER database is complete and accurate, so there is minimal risk of outcome misclassification.

Residual and unmeasured confounding by access to care and comorbid disease may influence our findings. In addition, there are other limitations of this study. First, the baseline survey did not discriminate between sigmoidoscopy/colonoscopy performed for colon cancer screening or symptoms. While it is likely most studies were performed for screening, there is undoubtedly exposure misclassification that likely attenuates the results. The findings of this study may not be generalizable to other populations since the VITAL cohort includes fewer smokers, is predominantly white, and is more highly educated than the general US population. However, although the response rate to the survey was only 21%, it is unlikely that selection bias could have affected our results because in a prospective design, subjects cannot participate jointly based on exposure and future (unknown) disease status.

## Conclusion

This study identifies several individual factors associated with stage at diagnosis of NSCLC, including an association between a screening activity and a lower risk of a diagnosis of advanced stage. This finding does not provide direct evidence that lung cancer screening is occurring though it raises that possibility. Our results may inform studies of healthcare disparities among patients with lung cancer. Furthermore, these results may prompt future studies of patient and clinician behaviors that may influence stage at diagnosis.

## Competing interests

The authors report no conflicts of interest with people or organizations that could inappropriately influence the work. The authors did not receive any outside assistance writing this manuscript.

## Note

The views expressed in this article are those of the authors and do not necessarily represent the views of the Department of Veterans Affairs.

## Authors' contributions

CS conceived of the study, designed and coordinated the study, performed the statistical analysis and drafted the manuscript. MG, DA, and MD participated in its design and coordination and helped draft the manuscript. EW designed and coordinated the study, and helped drafted the manuscript. All authors read and approved the final manuscript.

## Pre-publication history

The pre-publication history for this paper can be accessed here:

http://www.biomedcentral.com/1471-2407/11/228/prepub
